# Molecular status 36 months after TKI discontinuation in CML is highly predictive for subsequent loss of MMR—final report from AFTER-SKI

**DOI:** 10.1038/s41375-021-01173-w

**Published:** 2021-02-15

**Authors:** Johan Richter, Anna Lübking, Stina Söderlund, Kourosh Lotfi, Berit Markevärn, Anders Själander, Leif Stenke, Stefan Deneberg, Erik Ahlstrand, Kristina Myhr-Eriksson, Panayiotis Panayiotidis, Tobias Gedde-Dahl, Daniela Žáčková, Jiří Mayer, Ulla Olsson-Strömberg, Francois-Xavier Mahon, Susanne Saussele, Henrik Hjorth-Hansen, Perttu Koskenvesa

**Affiliations:** 1 Department of Hematology, Oncology and Radiation Physics, Skåne University Hospital, Lund, Sweden; 2 Department of Medical Sciences, University of Uppsala and Section of Hematology, Uppsala University Hospital, Uppsala, Sweden; 3 Department of Hematology, University Hospital, Linköping, Sweden; 4 Department of Hematology, University Hospital, Umeå, Sweden; 5 Department of Public Health and Clinical Medicine, Umeå University, Umeå, Sweden; 6 Department of Medicine, Karolinska University Hospital, Stockholm, Sweden; 7 Department of Hematology, Karolinska University Hospital, Stockholm, Sweden; 8 Department of Medicine, Faculty of Medicine and Health, Örebro University, Örebro, Sweden; 9 Department of Hematology, Sunderby Hospital, Luleå, Sweden; 10 First Department of Propedeutic Medicine, Laikon Hospital, National and Kapodistrian University of Athens, Athens, Greece; 11 Oslo University Hospital, Oslo, Norway; 12 Department of Internal Medicine Hematology and Oncology, University Hospital Brno and Masaryk University, Brno, Czech Republic; 13 Laboratoire Hématopoïèse Leucémique et cible thérapeutique, INSERM U876, Université Victor Ségalen Bordeaux 2, Bordeaux, France; 14 Medizinische Universitätsklinik Mannheim, Medizinische Fakultät Mannheim, Universität Heidelberg, Mannheim, Germany; 15 Department of Hematology, St Olavs Hospital, Trondheim, Norway; 16 Hematology Research Unit Helsinki, Helsinki University Hospital Comprehensive Cancer Center, Helsinki, Finland

**Keywords:** Chronic myeloid leukaemia, Phase II trials

## To the Editor:

Discontinuation of tyrosine kinase inhibitor (TKI) treatment for chronic phase (CP) CML patients with a deep molecular response (DMR) has entered standard practice. This is based on numerous clinical studies showing that ~50% of patients with DMR can stop TKI treatment without imminent disease relapse [1]. However, in most of these trials long-term follow-up has been limited [1].

In the largest stop trial performed so far, EURO-SKI, >800 patients were included and followed for 3 years after TKI stop [2]. Due to its size EURO-SKI represents an excellent base to study issues related to TKI stop in a structured manner. However, one limitation of the EURO-SKI study is that patients were only followed for 3 years. To investigate the incidence of later relapses, defined as loss of MMR ≥ 3 years after discontinuation, we performed a follow-up of 111 consecutive EURO-SKI patients in treatment-free remission (TFR) at 36 months, up to 6 years after TKI stop, in six countries. In the published interim analysis of 755 patients included in EURO-SKI, the TFR rate at 36 months was 49%. Thus, the follow-up data presented here cover about 30% of all the eligible TFR patients in EURO-SKI.

The detailed design of the EURO-SKI trial has been published [2]. In short, patients ≥ 18 years with BCR-ABL1-positive CP-CML with a typical BCR-ABL1-transcript (major: e13a2 and/or e14a2), receiving first-, or second-line TKI treatment due to toxicity to first-line TKI were included. Minimum TKI treatment duration was 3 years and minimum duration of DMR (MR4 or better) was 1 year. Patients with prior stem-cell transplantation, TKI failure, or having other malignancy were excluded.

Nineteen European centers from the Czech Republic, Finland, Germany, Greece, Norway, and Sweden participated in the AFTER-SKI follow-up of EURO-SKI. In agreement with each country’s public health code, the respective ethics committees approved the EURO-SKI protocol (ClinicalTrials.gov NCT01596114), as well as the AFTER-SKI follow-up.

Six years after inclusion in EURO-SKI data from consecutive patients in TFR at month 36 was collected: (1) patient alive or not, and if dead, cause of death, (2) number of RQ-PCRs performed per year, and (3) level of BCR-ABL1 (IS) at months 36, 48, 60, and 72. Whether the patient had continuously undetectable, occasionally detectable, or fluctuating levels of BCR-ABL1 was also captured. For patients who lost MMR, the time point, BCR-ABL1-value, restart of TKI, and reachievement of MMR and MR4 were reported.

The RT-qPCR testing of BCR-ABL1 in clinical practice after 36 months was assessed for all participating patients. During years 4–6 after TKI stop an average of 3.3 (range 0–6), 3.3 (range 1–5), and 3.2 (range 1–6) tests were performed per year, respectively. The variation in monitoring can probably partially be explained by a closer follow-up of patients with detectable BCR-ABL1 levels. However, for patients in MR4 the local or individual physician’s monitoring practice may have influenced the intensity of follow-up.

Three different patterns of BCR-ABL1 levels after TKI stop have previously been described by [3]. We used the same criteria to categorize patients that did not lose MMR during follow-up into three groups. Continuously undetectable BCR-ABL1, occasionally detectable values, or fluctuating levels of BCR-ABL1 (≥2 sequential detectable values) was found in 54%, 20%, and 26% of patients, respectively.

With a follow-up of 72 months, 12 out of 111 patients (10.8%) who were in TFR at 36 months, subsequently lost MMR (Supplementary Table 1). These relapses occurred between months 40 and 72 (median 51 months) after TKI stop. At relapse the BCR-ABL1 (IS) level was between 0.1 and 0.2% in all of these patients. The kinetics of relapse was slow with an ~1.5 fold rise of in BCR-ABL1 per month prior to loss of MMR differing from the rapid relapses seen during the first 6 months after TKI stop. Median duration of TKI therapy prior to TKI stop in the group who lost MMR during follow-up was 89 months compared to 92 months in the entire EURO-SKI cohort. Median duration of DMR prior to TKI stop in the relapsing patients was 43 months compared to 56 months for all the EURO-SKI patients. No progressions to AP or BC were observed but two patients died during follow-up due to causes unrelated to CML.

TKI therapy was restarted in all relapsing patients but one (Supplementary Table 1). This one patient lost MMR and then without restarting TKI therapy regained MMR. Since then the BCR-ABL1 level has fluctuated between MMR and MR4 now for >2.5 years. All patients who restarted TKI therapy regained MMR within 1–5 months (median 3 months) after restarting. All patients except one also regained MR4 within 1–5 months (median 3 months) after restarting TKI.

Status at 36 months appears highly predictive of later relapse as only 1 patient out of 98 in MR4 at month 36 lost MMR in the following 3 years (Table 1). This patient had a BCR-ABL1 value of 0.0087% at month 36 and then exhibited values between MMR and MR4 until relapse at month 54. Conversely 11 of the 13 patients not in MR4 at month 36 lost MMR during follow-up (Table 1).

**Table 1:** Molecular status at month 36 predicts later loss of MMR.

Status at month 36	Continued TFR m 36–72	Loss of MMR m 36–72	Total
MR4	97	1	98
Not in MR4	2	11	13
Total	99	12	111

In most trials of TKI discontinuation in CML follow-up has generally been limited to a few years and only occasional late relapses occurring ≥ 36 months after TKI stop have been reported [1]. A long-term follow-up of the pioneering STIM1 study was reported by Etienne and co-workers. In the cohort of 100 patients with median follow-up of more than 6 years no relapses occurring more than 2 years after TKI stop were observed [4]. However, the definition of relapse in STIM1 was quite different from the currently agreed loss of MMR at one time point. In STIM1, molecular relapse was defined more strictly as positivity of BCR-ABL transcript in a qRT-PCR assay confirmed by a second analysis that indicated an increase of one log in relation to the first analysis or loss of MMR at one point [5].

The risk of late losses of MMR several years after stopping TKI therapy has only been assessed in a structured way in one study. Recently, Rousselot et al. [6] published a long-term follow-up of the A-STIM. This single center study was the first major study to use MMR as relapse criterion. In all, 128 patients were included and 62 (48%) were in TFR at 3 years. Six of these relapsed subsequently between 3.5 and 6.4 years post TKI stop, corresponding to 4.7% of the included patients and 9.7% of those in TFR at 36 months.

The multicenter, multinational EURO-SKI study with the largest number of included patients forms an excellent base to investigate the issue of late relapses. We conducted a follow-up of 111 patients in TFR at 36 months and observed 12 relapses (10.8%) the subsequent 3 years. Considering the TFR rate at 3 years in EURO-SKI (49%) this translates into a 5.3% late relapse rate in the starting population, very much in line with findings made by Rousselot et al. [6] as mentioned above.

All of the relapses in our study were just above the MMR threshold (0.1–0.2% on IS), and evolved slower than early relapses after TKI discontinuation. This raises the question whether therapy must be reinstated directly after loss of MMR at a single time point in every patient. In at least of some of these cases confirmation of the loss of MMR with an immediate second BCR-ABL1 assessment might be feasible before discussing the pros and cons of restarting therapy with the patient. The one patient in our cohort who did not restart therapy further underlines this point. There may be patients who prefer continued close monitoring to restart of TKI, especially if they suffered from side effects of therapy. In an observational study from Italy four patients who lost MMR after TKI discontinuation did not resume treatment based on a shared decision between the patient and the physician [7].

In our opinion the most interesting finding of this study was that molecular status 36 months after TKI stop was highly predictive of molecular relapse. Only 1 out of 98 (1%) in MR4 at this time point lost MMR during the following 3 years. Not being in MR4 raised the risk to 85% (11/13 relapsed). Thus it may be possible to differentiate the intensity of continued molecular monitoring after 3 years depending on the molecular status. The ELN recommendations advocate continued measuring of BCR-ABL1 level every 3 months indefinitely [8]. However, it may be that those in MR4 require assessment of BCR-ABL1 only once or twice a year, while those not in MR4 should be monitored every 3 months continuously.

In summary, we show that late relapses after TKI discontinuation in CML do occur in ~10% of patients in TFR at 36 months. Molecular status at 36 months, not being in MR4, is highly predictive of subsequent loss of MMR.

## Supplementary information

Supplemental Table
