# Supplementary material for: Molecular status 36 months after TKI discontinuation in CML is highly predictive for subsequent loss of MMR—final report from AFTER-SKI
Source: Leukemia. 2021 Feb 15;35(8):2416–8. doi: 10.1038/s41375-021-01173-w (PMC8324471; doi:10.1038/s41375-021-01173-w)
Supplement: Supplementary file 1 — Supplemental Table [file 41375_2021_1173_MOESM1_ESM.docx]

**Supplemental table 1. Patients that lost MMR between month 36 and 72.**

| UPN | BCR-ABL month 36 | MMR  loss  (month) | BCR/ABL on IS  at MMR-loss | BCR/ABL on IS  prior to stop | Duration of TKI prior to stop (m) | Duration of MR4 prior  to stop (m) | Restart  of TKI (type) |
| --- | --- | --- | --- | --- | --- | --- | --- |
| FIN-001-006 | 0.07 | 45 | 0.18 | UD | 84 | 36 | Ima |
| FIN-001-019 | 0.09 | 40 | 0.18 | UD | 105 | 44 | Ima |
| FIN-001-020 | 0.02 | 52 | 0.11 | UD | 38 | 21 | Nilo |
| SWE-002-007 | 0.04 | 57 | 0.19 | 0.001 | 114 | 80 | Ima |
| SWE-006-002 | 0.017 | 60 | 0.15 | UD | 103 | 73 | Ima |
| NO-004-006 | 0.045 | 48 | 0.13 | UD | 159 | 86 | None |
| GR-001-008 | 0.013 | 46 | 0.103 | UD | 54 | 42 | Ima |
| CZ-001-007 | 0.057 | 50 | 0.13 | 0.0009 | 148 | 22 | Ima |
| CZ-001-015 | 0.0087 | 54 | 0.13 | 0.004 | 39 | 12 | Nilo |
| CZ-002-001 | 0.015 | 72 | 0.16 | 0.0027 | 94 | 25 | Ima |
| GE-009-005 | 0.038 | 45 | 0.11 | UD | 54 | 46 | Ima |
| GE-009-022 | 0.038 | 72 | 0.13 | UD | 51 | 44 | Nilo |
| **Median** | NA | 51 | 0.13 | NA | 89 | 43 | NA |
| **EURO-SKI** | NA | NA | NA | NA | 92 | 56 | NA |

UPN = unique patient number, UD = undetectable, NA = not applicable
